# Effect of trauma-informed care on hair cortisol concentration in youth welfare staff and client physical aggression towards staff: results of a longitudinal study

**DOI:** 10.1186/s12889-019-8077-2

**Published:** 2020-01-07

**Authors:** Marc Schmid, Janine Lüdtke, Claudia Dolitzsch, Sophia Fischer, Anne Eckert, Jörg M. Fegert

**Affiliations:** 1 Department of Child and Adolescent Psychiatry, University Psychiatric Hospital Basel, Wilhelm-Klein-Strasse 27, Basel, Switzerland; 2 Research Department for Child and Adolescent Psychiatry of the University of Basel, Basel, Switzerland; 3 Department of Child and Adolescent Psychiatry and Psychotherapy, University of Ulm, Ulm, Germany; 4 Neurobiological Laboratory, University Hospital Basel, University of Basel, Wilhelm Klein-Str. 27, 4012 Basel, Switzerland

**Keywords:** Client aggression, Hair cortisol, Trauma-informed care, Youth welfare

## Abstract

**Background:**

Professional caregivers working in child and youth welfare institutions are frequently faced with the complex mental health issues, emotional needs and challenging coping strategies of clients with cumulated traumatic experiences, leaving them prone to developing high levels of stress, burn-out and compassion fatigue. Trauma-informed care (TIC) is a milieu-therapeutic approach that aims to promote the self-efficacy and self-care of youth welfare staff by guiding them to a better understanding of their own and their clients’ stress symptoms and countertransference. Despite increasing efforts to implement TIC practices, and more widespread recognition of their value in youth welfare systems, there is a lack of studies evaluating the effectiveness of this approach. The aim of this study was to assess the effects of TIC practices in youth welfare institutions on both the physiological stress of staff members and clients’ physical aggression towards their caregivers. .

**Methods:**

Data was obtained from a longitudinal study investigating the effectiveness of TIC in 14 residential youth welfare institutions. Our sample consisted of 47 youth welfare employees (66.0% female) aged from 23 to 60 years (*M* = 37.4, *SD* = 10.4 years). Hair cortisol concentration (HCC) and occurrences of client physical aggression were assessed at four annual measurement time points (T1 to T4).

**Results:**

Participants in five institutions employing TIC practices (intervention group) showed significantly lower HCC at T4 than staff members from institutions who did not receive training in TIC (control group), indicating reduced physiological stress levels. At T4, the intervention group reported significantly less physical aggression than the control group.

**Conclusions:**

TIC might be a promising approach for reducing the emotional burden of employees and institutions should invest in training their staff in TIC practices. More research is necessary, to investigate the benefits and efficacy of TIC, both to youths and staff members, and to foster a better understanding of which specific factors may contribute to stress reduction.

## Background

Many children and adolescents in the youth welfare system have experienced traumatic stress. They were witness to, and/or themselves victims of, child maltreatment and neglect, domestic violence, or emotional, physical, or sexual abuse. It is estimated that up to 78% of children and adolescents cared for by the youth welfare system had been exposed to traumatic events and 58% had experienced multiple traumatic events [1]. Youths with a trauma history, particularly those living in residential care, have an elevated risk of mental health problems such as anxiety, depression, externalising disorders, substance abuse, or risk-taking behaviour [2, 3]. Moreover, these traumatic experiences can have long-lasting effects on the young person’s concept of self, cognitive control mechanisms and problem solving, relationships with others, and attachment to caregivers [4–6]. In order to account for the maltreatment experiences and trauma-related needs of this high-risk population, efforts have been made to implement trauma-informed care (TIC) practices in various psychosocial settings, especially youth welfare settings [7–9]. But TIC concepts should not be limited to residential group homes and youth welfare institutions [10]. They are relevant for all psychosocial settings, such as juvenile justice institutions [11, 12], special needs schools, child and adolescent, as well as, adult psychiatric settings [13], paediatric health care networks [14], shelters for the homeless, refugee centres (9), rehabilitation and detox centres [15] etc. A systematic review [16] of TIC literature on concepts with staff training reported 23 implementation and evaluation studies. The conclusion is, that TIC may improve clinical practice and can reduce trauma symptoms and the psychopathology of clients. Due to the high prevalence of traumatic life events in young people living in out of home placement and the need of placement continuity, some countries have realized countrywide implementation processes [17–19]. The challenge for youth welfare, juvenile justice, mental health institutions regarding the implementation process of TIC is that lies in the process of organization development which includes fundamental changes in attitudes and key processes and scrutinizes established institutional practice and structures [20, 21]. However, overall the implementation of TIC is associated with higher staff satisfaction [22].

Branson [11] describes, in his systematic review, different key variables of TIC. The interventions described are very close to the TIC concepts established in German speaking countries [23, 24]. These concepts share many of the characteristic approaches and ideas with concepts established internationally, especially on the level of interventions. The approach practised in German-speaking countries, but also some other concepts, focus on the administrative, professional and emotional support of the staff and constructive structures for inner and outer safety on the entire milieu-therapeutic ward.

TIC is a conceptual framework and milieu-therapeutic approach “that is grounded in an understanding of and responsiveness to the impact of trauma, that emphasizes physical, psychological, and emotional safety for both providers and survivors, and that creates opportunities for survivors to rebuild a sense of control, self-efficacy and empowerment” [25]. TIC conceptualises, and reframes, problem behaviour in the context of an individual’s traumatic experience(s), and involves anticipating and avoiding institutional and individual practices that could increase the risk of traumatic re-enactment [25]. Besides addressing the needs of traumatised individuals, TIC further aims to promote the self-efficacy and work related resilience of youth welfare staff, by guiding them to a better understanding of their countertransference and personal stress symptoms and by promoting their self-care [23, 26–28]. Continuous self-awareness and self-care may reduce stress and distress among social-service professionals, thereby enhancing work satisfaction and quality of care in the institution [29, 30]. The ability of staff members, who interact with severely traumatised children, to cope with stress and apply self-efficacy, are key factors for alternative correctional experiences, the reorganisation of trustful relationships and positive attachment representations (f. ex. [22, 24, 31, 32]).

Youth welfare employees are continuously exposed to the traumatic experiences and challenging emotional and behavioural coping strategies of their clients and are at increased risk for developing burnout and secondary traumatic stress [28, 30, 33]. Furthermore, traumatic experiences of the children and adolescents may increase the risk of escalating interactions and physical violence against youth welfare staff [34, 35]. Much of the abundant literature on the association between traumatic experiences and auto- aggression has come to the conclusion that traumatic experiences in childhood are a prominent risk factor for aggressive behaviour and conduct problems throughout the course of a survivors’ life [12, 36–43]. The close correlation could be explained, for example, by the misinterpretation of specific social interactions [44–48], model learning, deficits in implicit and explicit emotion regulation [49], especially the self-regulation of aggressive impulses, deficits in the ability to mentalise and be empathic [50] . Since adverse childhood experiences are associated with an impaired ability to regulate or tolerate negative emotions, as well as externalising types of behaviour, affected youths may find themselves relying on counterproductive and detrimental coping strategies, such as opposition, aggression or delinquency when confronted with challenging emotions and situations (e.g., trauma triggers) [36–39]. Therefore, it is not surprising that frequent exposure to client aggression is a common reality in the professional life of social workers [34, 35, 51]. Alink et al. [34] found that 81% of youth residential care staff experienced client aggression, and about half of them reported physical aggression, within the last year. Another study reported that 91% of youth welfare staff experienced at least one type of verbal and physical aggression, with 53% reporting verbal threats and 24% experiencing physical violence in the past three months [35]. A central aim of TIC is to ensure internal and external safety for both social workers and their clients, including the prospective prevention of violent behaviour [35]. However, the question whether implementation of TIC is associated with a reduction in physical violence against youth welfare staff has barely been investigated. A review on effective strategies for implementing TIC in youth psychiatric and residential treatment settings concluded that TIC might lead to a decrease in client and staff injury rates [8]. However, more longitudinal studies are needed, to demonstrate that TIC significantly improves client and provider safety.

While TIC has proved beneficial to social functioning, emotion and behavioural regulation of children and adolescents served by the youth welfare system [27], studies on the effectiveness or benefits of TIC on staff level are still lacking. One study reported that trauma-informed self-care strategies may increase compassion and job satisfaction, as well as reduce symptoms and burnout among youth welfare staff [28]. Although improving the stress management of employees is a central aim of TIC [23, 52], no previous studies have investigated whether TIC has an effect on stress levels among youth welfare staff.

When the human body is under acute stress, the hypothalamic-pituitary-adrenal (HPA) axis releases the glucocorticoid cortisol, a central biomarker of stress that enables effective coping with stressors via the regulation of basal processes, such as inflammatory and immune responses [53–55]. Cortisol is traditionally measured in the blood, urine, or saliva, but these measurements only reflect short periods of time. In contrast, obtaining hair cortisol concentration (HCC) is a promising approach to measuring long-term cortisol release (for a review see [56]). A recent meta-analysis concluded that HCC is a valid indicator of stress. Individuals with chronic stress exhibited a 22% higher HCC, and among those with ongoing stress, the increase in HCC was around 43% [56]. Since TIC aims to reduce stress levels among professionals working with traumatised clients [23, 26–28], it is of great interest to compare whether there are long-term differences in HCC between youth welfare employees from institutions with and without TIC practices.

Despite growing implementation efforts of TIC practices and recognition of their value in youth welfare systems, there is a lack of studies evaluating the effectiveness of this approach [27]. So far, this is the first longitudinal study investigating the influence of TIC on HCC and physical aggression towards youth welfare employees.

The aims of our longitudinal study were twofold. First, we wanted to examine whether the occurrence of physical violence towards youth welfare staff differs between staff members receiving training in TIC practices and those providing the usual care. Second, we aimed to investigate the longitudinal course of HCC among youth welfare staff with training in TIC practices and providing usual care. Our research aimed to answer the following questions:

1. How high is the prevalence of physical aggression towards youth welfare staff?
2. Do youth welfare employees who received training in TIC and those providing usual care differ with respect to the prevalence of physical aggression as assessed at four time points?
3. Do the two groups differ with respect to HCC over the course of the study?

## Methods

### Participants

A total of 142 youth welfare employees participated in the study. Overall, 95 participants had missing data with respect to HCC and physical aggression and were therefore excluded from the study (see Table 1 for missing data with respect to HCC and physical aggression across the four measurement time points).

**Table 1:** Missing data with respect to HCC and physical aggression across the four measurement time points

Variable	Any missing data			
	T1 N (%)	T2 N (%)	T3 N (%)	T4 N (%)
HCC	12 (8.5)	21 (14.8)	56 (39.4)	83 (58.5)
Client physical aggression	8 (5.6)	14 (9.9)	57 (40.1)	83 (58.5)

Note. HCC = Hair cortisol concentration

Reasons for missing data were turnover, maternity leave, medical leave, a change of job within the institution, job loss and retirement. Our analysis included 47 participants who had complete HCC and physical aggression datasets for all four measurement time points (intervention group [IG]: *n* = 18; control group [CG]: *n* = 29).

Table 2 shows the descriptive statistics for sociodemographic variables, occupation and professional experience. Mean age of the final sample was 37.4 years (*SD* = 10.4 years), and 66.0% of the population were female. Groups did not differ significantly with respect to age, but the CG had a significantly higher female proportion (IG: 44.4%; CG: 79.3%; 휒^2^ [1, *N* = 46] = 6.01, *p* = .01). The majority (85.1%) of participants were social education workers or social education workers in training with an average of 8.96 years (*SD* = 9.11, range = 0–37 years) of professional experience in residential youth welfare institutions and having worked in the present institution for an average of 3.96 years (range = 0–18 years). Groups were comparable across occupation and professional experience. The attrition was controlled. The statistical analyses show no significant differences between the analyzed sample and rest of the sample in regard with demographic variables like age, gender, and professional experience (*p* = .187 to *p* = .396), as well as physical aggression towards youth welfare staff (t1-t4; *p* = .235 to *p* = .511), and Cortisol-level (t1-t4; *p* = .066 to *p* = .239).

**Table 2:** Sociodemographic sample characteristics

Variable	Sample	Control group (*n* = 29)	Test statistic	*p*
	Intervention group (n = 18)			
Age M (SD)	35.33 (8.82)	38.66 (11.18)	*T (df)* −1.06 (45)	.291
Gender n (%)				
Male	10 (55.6)	6 (20.7)	χ^2^ *(df)* 6.01(1)	.014
Female	8 (44.4)	23 (79.3)		
Occupation n (%)				
Social worker	16 (88.9)	24 (85.7)	*F*n/a	.564
Teachers, psychologists, others	2 (11.1)	4 (14.3)		
Professional experience in years M (SD)	6.80 (6.09)	10.40 (10.53)	*U* 276.500	.437
Professional experience in the current institution in years M (SD)	3.12 (2.45)	4.48 (4.88)	*U* 260.500	.598

Note. *M* mean, *SD* standard deviation, *df* degrees of freedom, *F* Fisher’s exact test, *n/a* not available, *U* Mann-Whitney-U-Test

### Procedures

We obtained the data from a government-funded exploratory model project investigating the effectiveness of TIC in 14 residential youth welfare institutions, of which 5 institutions received implementation of TIC, conducted in the German speaking part of Switzerland between 2012 and 2015. We contacted all residential youth welfare institutions approved by the Swiss Federal Office of Justice, (SFOJ) and invited them to participate in the model project. One recommendation of the SFOJ was to include different categories of institutions in this project and open it to all institutions approved for by the SFOJ. Due to this recommendation, co-educative and institutions for male and female adolescents only were included in our study as well as one institution with a special needs school and one with an integrated job training programme.

An advisory board, consisting of members of the SFOJ, independent experts and the project team, who later conducted the TIC trainings, selected suitable institutions among those, who were endorsed to participate in the project. We ran a naturalistic control group design and allocated the institutions to either of the two groups (IG or CG), carefully matching them in terms of comparable qualities of care (e.g., staff education, resident-staff-ratio, referrals). The selected institutions accommodate children, adolescents, and young adults between 7 and 25 years of age, characterised by high levels of traumatic experiences and clinically relevant internalising and/or externalising behaviour, with over a third of them having a criminal record or with symptoms of severe deficits in social behaviour [57]. 80% of the children and adolescents in the institutions reported traumatic life events in the Childhood Trauma Questionnaire (CTQ) and 76% reached the clinical cut-off in the Child behaviour checklist (CBCL Total Score). 15% were referred by penal law entities, 67% by civil law /child protection services and 18% attended special schools and working training voluntarily. The children in IG and CG do not differ significantly regarding age, psychosocial burden, traumatic life events, CBCL, Maysi-2 Scores.

We used a longitudinal study design, to prospectively investigate changes in HCC and physical aggression towards youth welfare staff from each institution at four annual time points (T1 to T4). Data collection comprised self-report questionnaires on sociodemographic variables and experience of aggression at the workplace, as well as hair samples for cortisol analyses. All participants received full information on the study aims and procedures and all gave written informed consent. The leading Ethics Committee of Northwest and Central Switzerland (EKNZ), as well as the Ethics Committees of the Cantons of Bern, St. Gallen, Aargau, Zürich, and Ulm (Germany), approved the study.

### Implementation of TIC practices in youth welfare institutions

TIC aims to transform an entire system of care by embedding an understanding of the dynamics and impact of trauma on youths and by creating a safe environment and culture of care, trust, choice, and collaboration [58, 59]. In order to create such an atmosphere, it is crucial to address the security and self-efficacy of the residential staff as well as reorganise some key institutional processes. It is necessary that professionals on all levels of the organisation, including Management, are committed to changing their existing attitudes and practices. Therefore, the management staff and counsellors underwent a specific training in organisational development, supervision skills, and burnout prevention. Implementation of TIC requires a long-lasting commitment of the institutions to allocate resources and building capacity, to fully train the staff with respect to values and principles of TIC, e.g., knowledge of neurobiological and behavioural sequelae of trauma, awareness of trauma triggers, intervening in a trauma-sensitive way, and attention to self-care in response to working with traumatised clients [23, 57]. Apart from intensive training, the uptake of TIC practices requires ongoing supervision as well as sufficient time for the transfer of knowledge and the consolidation of new strategies into institutional practice.

For three consecutive years, experienced professionals conducted advanced training to implement and support TIC in youth welfare institutions (six 3-day trainings for the management and counsellors, eight 2.5-day trainings for the youth welfare staff). The training was mandatory for all the employees in the participating institutions. New employees in the intervention settings were included in the ongoing implementation process and received the respective training. In between trainings, institutions received ongoing supervision in implementing a trauma-informed philosophy and services, debriefing on critical incidents and support in promoting an organisational culture of well-being, permanency, safety, care, and respect towards clients and co-workers [60].

At the third time point, i.e. by the end of the last training block, all key procedures must be fully implemented in the institutions on all levels of management. The implementation process includes new strategies for the supervision of challenging interactions [61] between clients and staff, psycho educational sessions and so-called resilience hours in a one-to-one situation. The focus is on good, joy-filled interactions and includes some training in emotion regulation, mindfulness, mentalization and social problem solving skills. Furthermore, institutions should revise, and if necessary improve, their key operational procedures (rules, documentation, admission, treatment planning), with a special focus on TIC.

The implementation process leads to a TIC concept with the following characteristics, which must be implemented by the end of the last training (t3):

- Concept of an internal (relationship, self-efficacy) and external (rules, crisis plan, room concept) safety place for staff and clients.
- Special types of case supervision (at least once per month) – including interaction analysis with a focus on security, self-efficacy and stress reduction of the staff [61].
- Psychoeducation with every client about the link of adverse life events with emotion and anger regulation problems, dissociation and self-efficacy.
- Regular (at least once per month) one to one situations with clients, with a special focus on positive interaction and resilience skills.
- Group feedback sessions (at least one per month) with a focus on developing a positive peer culture.
- Reflection of institutional procedures with regard to TIC concepts and attitudes such as transparency, participation, good reason, respect for the individual needs of every client etc.

At four annual time points (T1 = baseline, T2 = after 12 months, T3 = after 24 months, T4 = after 36 months), the youth welfare staff completed several questionnaires covering sociodemographic variables, perceived collective efficiency, sense of coherence, self-care, job satisfaction, personal boundary violation, as well as symptoms of post-traumatic stress, secondary traumatic stress, and burnout. Furthermore, hair samples were collected for hair cortisol analyses, and extensive qualitative interviews were conducted to evaluate the implementation process of TIC. The implementation process completed after 3 years. Over the course of the study, none of the providers in the control group received training in TIC, however providers in the control group received training in TIC after the study was completed.

### Measures

#### Violent behaviours towards youth welfare staff

A self-developed survey about personal boundary violations at the workplace [62] assessed verbal and physical aggression by children and adolescents towards employees, aggression among children and adolescents, and self-injuring or suicidal behaviour of children and adolescents during the past three months. To address our research questions, only the items relevant to physical aggression by children and adolescents towards employees were analysed. Participants were asked to indicate whether they had experienced physical aggression by clients in the past three months, e.g., getting kicked, getting bitten, or having objects thrown at them.

#### Hair cortisol analysis

Hair was collected from the posterior vertex region [63]. Strands of hair (1.5 cm long) adjacent to the scalp were analysed. Given an average hair growth rate of 1 cm/month [64] the examination of a 1.5 cm hair segment allowed the assessment of cumulative cortisol secretion over the previous six weeks. Hair cortisol was extracted as described by Gao et al. [65]. Cortisol levels were determined using a commercially available, high-sensitivity (analytical sensitivity 0.007 μg/dL) salivary cortisol enzyme immunoassay kit (Salimetrics Europe, UK) according to the manufacturer’s protocol. The intra-assay and inter-assay coefficients of variation of this assay are below 9%. Samples were analysed in duplicate, and mean values of respective measurements were used in statistical analyses. All measures were done in a blinded fashion. Values are expressed as pg cortisol/mg hair.

#### Data analysis

Descriptive statistics and group differences were calculated for the IG and CG. Categorical variables were analysed using Pearson’s Chi-square or Fisher’s exact test, and continuous variables were computed with Student’s t-test. Hair cortisol data were positively skewed and therefore log-transformed. A one-way repeated-measures analyses of variance (ANOVA) with HCC as within-subject factor and group (IG, CG) as between-subject factor was conducted for testing differences in HCC. Univariate ANOVAs were further carried out, to separately compare HCC in the two study groups. Age and gender were included as additional factors to control for possible confounding effects. Means and standard deviations of HCC are provided in log-transformed units (pg/mg). Statistical analyses were performed using SPSS for Windows, version 24.

## Results

### Client aggression towards youth welfare staff

Table 3 shows the prevalence of client physical aggression towards youth welfare staff over the four time points. Across all four time points, 13.6 to 22.2% of the total sample experienced physical aggression by clients. Youth welfare staff in the CG were significantly more often exposed to physical aggression at time 4 than youth welfare employees in the IG (CG: 24.1%; IG: 0.0%; *p* = .02, Fisher’s exact test). No significant differences between the groups were found for the remaining time points.

**Table 3:** Prevalence of client physical aggression towards youth welfare staff in the intervention and control groups

	Intervention group (*n* = 18)^a^	Control group (*n* = 29)^a^	Total (*n* = 47 ^a^)		
	%			χ^2^ (df = 1)	*p*
T1	16.7	25.9	22.2	–	.363 F
T2	17.6	11.1	13.6	–	.426 F
T3	20.0	20.7	20.5	–	.641 F
T4	0.0	24.1	15.2	–	.029 F

Note. F = Fishers exact test, ^a =^ Total Ns varied marginally due to missing data

### Hair cortisol analyses

**Fig. 1:**
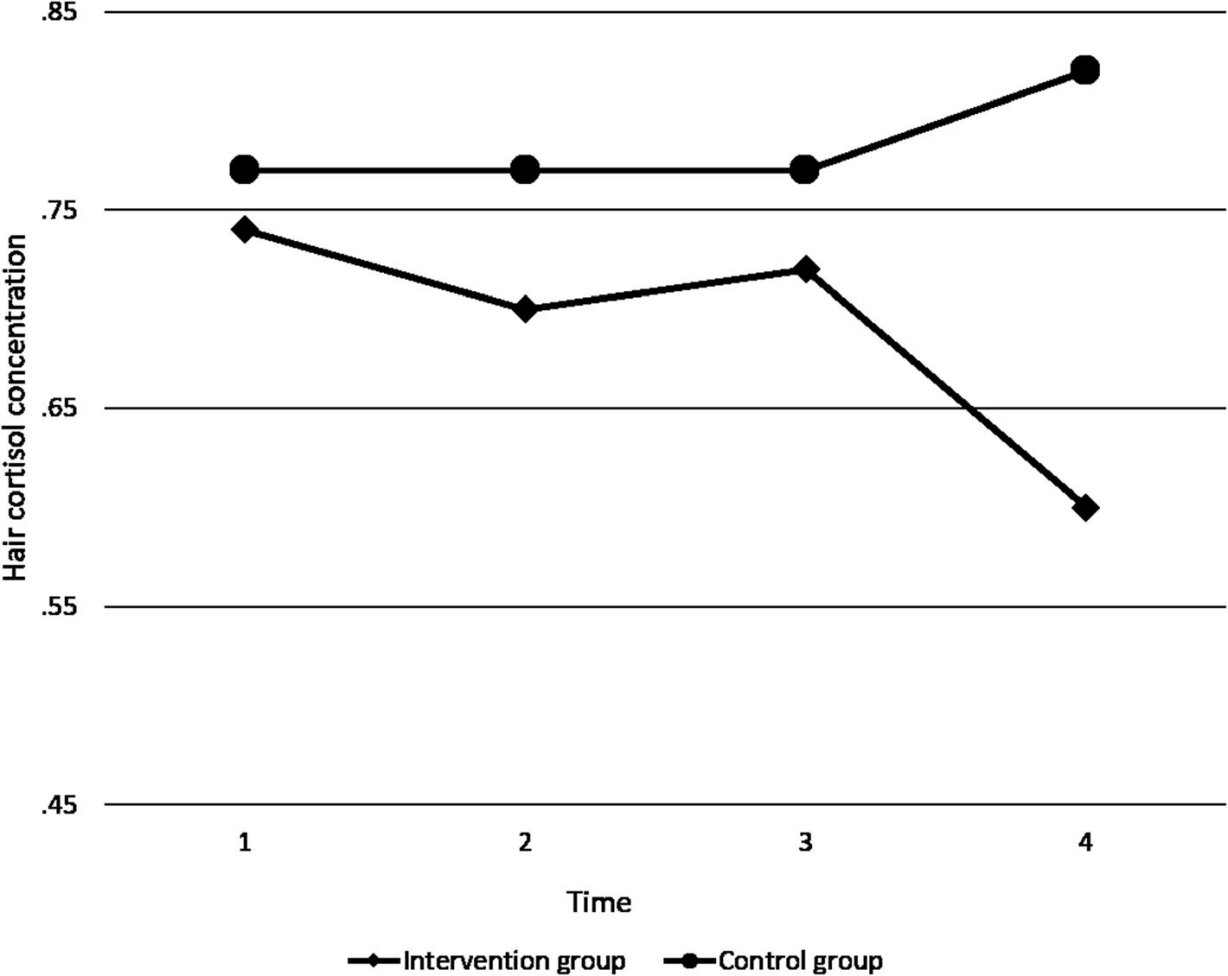
Hair cortisol concentration across the four time points for the intervention group and the control group

Figure 1 shows the log-transformed HCC data at the four sampling time points in the IG and CG. A one-way repeated-measures mixed ANOVA revealed no significant main effect of HCC (F [3, 46] = 1.846, *p* = .142, η2 = .041); however, the interaction between HCC ˟ group was significant (F [3, 46] = 3.365, *p* = .021, η2 = .073). Table 4 shows the means and SD for HCC in the two study groups. One-way ANOVAs revealed a significant difference in HCC between groups at T4 (F [1, 46] = 11.017, *p* = .002, η2 = .610), with the IG showing lower HCC than the CG (see Table 4).

**Table 4:** Comparison of log-transformed HCC at the four time points between the intervention and the control group

	Intervention group M (SD)	Control group M (SD)	F (df)	*p*	η2
T1	.74 (.04)	.77 (.03)	.017	.898	.001
T2	.70 (.05)	.77 (.04)	.069	.794	.003
T3	.72 (.05)	.77 (.04)	.400	.530	.020
T4	.60 (.06)	.82 (.04)	11.017	.002	.610

Note. HCC = hair cortisol concentration

## Discussion

We investigated the impact of TIC practices on HCC and occurrence of client physical aggression among youth welfare staff in a longitudinal study. Our results showed a significant difference in HCC and client physical aggression between the two groups at T4, with the IG showing lower HCC as well as reporting less physical client aggression than the CG. These results indicate that after implementation of TIC, youth welfare staff in these institutions showed significantly reduced stress levels and experienced fewer client physical aggression compared to staff who did not receive training in TIC. The significant reduction after T4 indicates that training and knowledge of psycho-traumatology are not enough to change institutional practice and reduce the stress level of staff [22, 66] and that such implementation processes take time and the allocation of resources [20]. It appears necessary to develop and implement TIC key procedures in those institutional processes and structures focusing on client and staff safety and self-efficacy in interactions, towards a kind of supply chain in which the management supports staff and staff support clients.

Previous studies highlighted the benefits of TIC practices such as increased compassion satisfaction and reduced symptoms of burnout among youth welfare staff [28]. Our study is the first to show a decrease in physiological stress among participants working in institutions with a TIC approach. One reason for the stress decrease might be the focus of TIC on establishing emotional safety by promoting self-care on a personal and institutional level [23, 25]. For instance, trauma-informed institutions may provide structures that allow their employees to institute support groups after critical incidents or difficult interactions, with the possibility to reflect on one’s own feelings and motives in order to foster and maintain mental hygiene, coherence, mindfulness, and resilience of the staff [52, 60].

We found rates of physical aggression towards youth welfare staff comparable to those reported in the literature, which illustrates that client aggression is a pressing concern that needs to be addressed [34, 35]. However, it is noteworthy that at the last measurement time point, participants in the IG experienced significantly less client physical aggression than those in the CG. Russell et al. [67] showed that TIC is associated with a decline in staff injury rates. Exposure to client aggression is associated with impaired physiological and emotional well-being and may have implications for work satisfaction and quality of care [46, 68, 69]. Of course, this is a bidirectional association, which could lead to either a positive or a vicious circle: boundary violations against staff lead to insecurity and low self-efficacy, this induces reduced pedagogic presence and positive interactions, which can, in turn, enhance the risk of new boundary-violations [70].

Therefore, it is crucial to establish prevention and intervention standards to ensure the physical safety of employees. Furthermore, from a perspective that regards aggression as a failure to regulate emotions in the face of threatening or frustrating situations [60, 71] and through a trauma-sensitive lens, it might be helpful to create an atmosphere of shared decision-making with youths and thus promote their self-regulation and coping skills [72]. The staff’s ability to regulate and contain their own emotions in highly stressful interactions is of equal importance in order to recognise and adapt to the young people’s needs [73]. As noted above, client physical aggression is highly prevalent in residential youth welfare institutions and may be seen as an innate and unavoidable occupational hazard [35, 73]. Therefore, the staff should work within the framework of institutional structures that encourage communication and the sharing of their concerns and highly emotional experiences in these challenging situations [74].

The general mental health needs of children and adolescents cared for by the youth welfare system have been extensively studied [1–3], whereas knowledge about the professionals’ experiences and psychological impact of this challenging area of work is limited. McElvaney and Tatlow-Golden [75] report that professionals working in the care and youth justice system describe themselves as feeling helpless, frustrated and incompetent in the face of the complex mental health needs of their clients. The authors conclude that the staffs own psychological response mirrors the traumatic response of their clients thus, the staff members feel traumatised themselves, thereby possibly contributing to further client traumatisation. High rates of traumatic stress and/or compassion fatigue are common among youth welfare staff and may lead to job burnout, work withdrawal, and turnover [76–78].

Three years after the beginning of the study, when the TIC approach was fully adopted by the institutions, participants who received training in TIC had significantly reduced HCC compared to the control group. Moreover, TIC-trained study participants’ experienced significantly less physical client aggression than those who did not receive TIC training at T4. Training of individuals to support the adoption of TIC may be time-consuming and demanding and requires a long-term commitment of the institutions and their employees. However, our study suggests that implementing a TIC approach may be beneficial in the long run. Employees who feel less stressed and experience fewer physical assaults may be able to offer a better quality of care for their clients and remain in their jobs for longer, thereby fostering stability in the institution.

Several limitations of the current study have to be considered. As the data was taken from an exploratory study with longitudinal design, the final sample size was small, which might have precluded the ability to identify group differences. The complete set of measurements at the four annual time points was available for only 33% of the study population, which indicates a large participant drop-out rate due to turnover or other reasons. However, we have decided against an imputation of the missing values because participation in the entire implementation process is crucial for evaluating the effectiveness of TIC. High rates of staff turnover are a common problem in social services [76–78] and might have contributed to the small sample size in our longitudinal study. However, we controlled the attrition regarding different variables and could not find significant differences regarding psychosocial variables, hair cortisol concentration and burn out risk. The gender differences between IG and CG could be also a relevant limitation. Some studies show, that female staff is at higher risk of boundary violations and sexual harassment at in the workplace [79, 80]. We had very limited possibility to control and specify the correlation between violence against staff and gender in this small sample (we found no difference regarding hair cortisol concentration, TIC and boundary violations *p* > .175). It will be necessary to prove the effect of gender in studies with greater samples that take into account the effect of gender on boundary violations and hair cortisol concentration, even if we could not observe such an effect in our study.

Notably, large sample sizes in longitudinal studies involving neurobiological measures are rare and difficult to obtain. To the best of our knowledge, this is the first longitudinal study assessing neurobiological variables in the youth welfare system.

A further study limitation was the uncertainty on whether the TIC concepts were implemented in a uniform way by the participating institutions. Since all institutions had their established concepts, the degree of adherence to TIC principles (i.e., frequency of performing the interventions, quality control) may have differed. Given that institutions had unequal resources at their disposal for the implementation of TIC, and that the TIC approach tried to standardise highly heterogeneous concepts, employees at individual institutions may have encountered variable stress levels. It is possible that new employees in the intervention group have had training in TIC in previous jobs, however there was no cross-over of employees and the institutions participating in the study. Finally, since the experience of client aggression was based on self-reports, a certain report and recall bias cannot be excluded.

## Conclusion

Findings from the current study have important clinical implications. Our results suggest that TIC practices can successfully reduce physiological stress and client physical aggression among youth welfare employees working with traumatised children and adolescents. Most notably, we used a biological measure of stress instead of subjective stress ratings, to assess the physiological changes after implementation of TIC practices more accurately. The measured decrease in stress levels among the staff might be associated with the core principles of TIC, such as fostering and maintaining mental hygiene, coherence, mindfulness, and resilience. By implementing operational procedures that guide the staff to a better understanding of their own stress symptoms and promoting self-care, they might be better equipped to recognise and adapt to the young people’s needs and to avoid traumatic re-enactment. Therefore, we suggest that institutions should invest in training their staff in TIC practices and aggression de-escalation techniques.

Future research is needed to evaluate the effectiveness of TIC in larger samples and other populations (e.g., child and adolescent psychiatry, forensic units, closed juvenile justice settings, settings for adults, homeless people etc.) – TIC concepts are not limited to residential care or child and adolescent psychiatric settings, they have the potential to benefit practically all psychosocial settings. To what extent the results of these studies are transmittable to other psychosocial settings should be examined because, of course, boundary violations and high levels of stress in staff members are relevant in nearly all psychosocial settings.

It is likely, that the consistent focus on the staff and the reduction of staff stress levels to enhance the capacities for correctional client-staff relationships, with lower arousal in the whole interaction, is the innovative aspect that differentiates our Model [24, 31] from other TIC concepts (overview [11]).

In addition, it would be of interest to investigate which component of TIC is associated with the most marked decrease in physiological stress reactions. Potential additional benefits of TIC for employees and their clients should also be studied, and neurobiological changes induced by psychosocial interventions should be more fully understood. However, to the best or our knowledge, there are only few studies targeting individuals in residential care (e.g. [81]). Our study shows that neurobiological research is feasible in this field and offers new insight into physiological changes that accompany TIC.

## Data Availability

The datasets used and analyzed during the current study are available from the corresponding author on reasonable request.

## Abbreviations

ANOVA: Analyses of variance
CBCL: Child behaviour checklist
CG: Control group
CTQ: Childhood trauma questionnaire
DF: Degrees of freedom
EKNZ: Ethics Committee of Northwest and Central Switzerland
F: Fisher’s exact test
HCC: Hair cortisol concentration
IG: Intervention group
M: Mean
n/a: Not available
SD: Standard deviation
SFOJ: Swiss Federal Office of Justice
TIC: Trauma-informed care
U: Mann-Whitney-U-Test
